# Gravity Deprivation: Is It Ethical for Optimal Physiology?

**DOI:** 10.3389/fphys.2020.00470

**Published:** 2020-05-08

**Authors:** Jack J. W. A. van Loon, Patrick Cras, Willem H. A. C. M. Bouwens, Willemijn Roozendaal, Joan Vernikos

**Affiliations:** ^1^Department Oral & Maxillofacial Surgery/Pathology, Amsterdam Movement Sciences & Amsterdam Bone Center (ABC), Amsterdam University Medical Center Location VUmc & Academic Center for Dentistry Amsterdam (ACTA), Amsterdam, Netherlands; ^2^Faculty of Medicine & Health Sciences, Translational Neurosciences, University of Antwerp, Antwerp, Belgium; ^3^Department of Neurology, Antwerp University Hospital, Edegem, Belgium; ^4^Institute Born-Bunge, University of Antwerp, Antwerp, Belgium; ^5^Faculty of Law, Social Law, VU University Amsterdam, Amsterdam, Netherlands; ^6^Thirdage Health, Culpeper, VA, United States

**Keywords:** microgravity, pathology, ethics, chronic accelerations, artificial gravity, large diameter centrifuge, countermeasure, treatment

Probably a question nobody ever asked. It goes without saying that one takes care of persons for whom you are responsible especially when those persons totally depend on you. Papers in Frontiers, like in a recent special issue on “Gravitational Physiology, Aging, and Medicine” (Goswami et al., 2019) in Integrative Physiology but also in “Environmental, Aviation, and Space Physiology” identify various issues directly related to the lack of gravity and efforts to define countermeasures to possibly prevent pathologies. Also the recent papers by Trudel et al. (2019) regarding spaceflight related anemia or the works by Marshall-Goebel et al. (2019) showing in-flight thrombosis clearly illustrate our hiatus in the task of taking care when it comes to astronauts' and cosmonauts' health.

So, do we really take the best care of our fellow humans on their extraterrestrial travels? Based on the work mentioned above and the recent review by Stepanek et al. (2019), it is time to raise such health-ethics' related questions, in particular with respect to astronauts living and working in microgravity. Is it ethical to deprive astronauts of gravity?

Yes, astronauts are provided with food and oxygen, they are working in a cozy short-sleeve environment, they can drink water *ad lib* and call their loved ones at will. However, especially since Skylab in the 70s, it became clear that e.g., several bone parameters decreased significantly in crew members (Bikle and Halloran, 1999), later quantified by DEXA for Shuttle and International Space Station (ISS) crews to an average rate of 1% or more per space/month (Lang et al., 2017). And although the crew has a strict in-flight training protocol, bone parameters stay below pre-flight/pre-microgravity values even 1 year after return to Earth (Vico et al., 2017).

Some 50 to 75% of highly trained astronauts report suffering from the Space Adaptation Syndrome (SAS) in their first days of flight (Waldrop, 1982). Others experience some combination of headache, malaise, lethargy, anorexia, nausea, vomiting, and gastric discomfort during the first few hours or even days in microgravity, despite the use of various drugs (Zhang and Hargens, 2017). Most venture capitalists considering commercial space flight should probably think twice before investing in a business model where there is a high probability that the majority of their potential clientele might get sick in the first couple of days of their pricy space trip. To address this issue, a sufficient level of gravity may be provided by a large diameter rotating space hotel.

For some years crew members began reporting vision deficiencies. This phenomenon, first described by Mader and colleagues as VIIP (Visual Impairment due to Intracranial Pressure) (Mader et al., 2011) and now termed Spaceflight Associated Neuro-ocular Syndrome (SANS), urged ISS partners and especially NASA to identify a possible course of action. Recent reports argued that very significant changes in brain morphology, in particular long duration flights of astronauts (Roberts et al., 2017) and cosmonauts (Van Ombergen et al., 2018), might be associated to SANS. Although no clear cause was identified, one of these might be the high level of CO_2_. The initial ppCO_2_ levels in ISS started at 7.6 mm Hg (Law et al., 2014), some 25 times higher than we have on Earth! It was later lowered to 5.3, and because of the possible contribution of this hypercapnia to SANS, again quickly lowered to 4.0. Law et al. (2014) recommended a ppCO_2_ level of 1.97 mm Hg since this would keep the risk of headache to below 1%, a standard threshold used in toxicology and aerospace medicine. Note the average ppCO_2_ on Earth at sea level is around 0.3 mm Hg. One wonders why space agencies never reacted like this when it came to surviving microgravity.

Space agencies are engineering entities, created to develop and apply space related hardware and technology. Although their main focus is to keep the crew alive and safe, long duration flight health issues have not been fully identified and understood. As is clear from the carbon dioxide problem but also from the less than effective countermeasures, there is more to human health than keeping the crew alive.

In the various definitions for Life Support Systems used by space agencies such as NASA (U.S.A.) or the European Space Agency (ESA), for either physicochemical or biological systems, surprisingly there is no mention of gravity as an essential element for maintaining a healthy environment, while oxygen or humidity are.

In order to counteract the deleterious effects of gravity deprivation, astronauts spend hours of valuable crew time exercising each day. However, exercise is not tantamount to gravity, nor do we really know how effective it is. We have never flown an astronaut that did not exercise and therefore do not know how much worse it would be. Even assuming that exercise may indeed be partially beneficial, it generates other problems, such as increased core body temperature while training in microgravity (Stahn et al., 2017) and possibly increased intracranial pressure due to high loads during resistive training (Dickerman et al., 2000; Stenger et al., 2017).

Muscular and cardiovascular deconditioning are believed to be mostly addressed by exercise countermeasures. The alternative countermeasure of Lower Body Negative Pressure (LBNP) was found to be only 55% effective in the case of venous blood flow stasis (Marshall-Goebel et al., 2019). Yet, other issues remain unsolved such as impaired cognitive performance, renal stones, SANS, reduced immune sensitivity, loss of quality and duration of sleep, low back pain and osteopenia, as well as post-flight balance and coordination issues, orthostatic intolerance or spinal compression with intervertebral disk damage (Barger et al., 2014; Yaqub, 2015; Stepanek et al., 2019). These are believed to be due to inadequate body gravity loading in space.

When it comes to meeting the necessary gravity requirements for the health and safety of astronauts, space agencies should go beyond arguments of flight complexity and costs. What is the price of health and safety? Systems for large radius chronic centrifugation should form a serious part of their implementation plans for space exploration. It is technically feasible to have large rotating spacecraft (Joosten, 2007; Paloski and Charles, 2014; Hall, 2016; Martin et al., 2016). Ground based devices could be used to develop specific requirements, such as identifying minimum gravity and radius thresholds, while, as an add-on, treating contemporary diseases on Earth such as obesity and aging (van Loon et al., 2012). The resources required for an in-flight infrastructure are an investment in crew health and well-being and as shown by Joosten (2007) the additional cost in particular for configurations would only be around 5% supplementary structural or propellant mass. Physiological, psychological and social well-being should be an integral part of space station designs. It is unethical, life and mission threatening to withhold gravity from human beings just as the denial of oxygen would be.

As such, gravity should be regarded an integral part of a space station Life-Support System, just as regulated oxygen, humidity, carbon dioxide or temperature is. The exact requirements for the minimal g-profile for adequate to optimal Earth-like physiology in space are unknown and must be established (National Research Council, 2018). The preferred solution is a very large diameter rotating system, with a diameter of some 150 m (van Loon et al., 2012) or smaller at 50 to 110 m (Globus and Hall, 2017). Though one could start with a short arm on-board centrifuges but such systems would have problems of their own like large body g-gradients, not exposing the whole body including the vestibular system (Fuller et al., 2002; Levasseur et al., 2004; Ogoh et al., 2018) to functional gravity. it is to be expected that short-arm centrifuges will not generate the foreseen optimal treatment. A short arm system would also not decrease the valuable crew time spend on exercise. On the other hand, besides providing a 1 g countermeasure, a large rotating spacecraft could also be used to discover the long term effects of partial gravity in a relatively safe Lower Earth Orbit (LEO) in preparation for Moon and Mars explorations before being confronted with the unknown effects of chronic partial Mars and Lunar gravity.

Space agencies, as employers, carry an obligation to provide a safe and healthy working environment for their employees. Consequently, from a labor-legal point of view astronauts should be provided with the necessary means to work in a healthy environment, including the multi-system countermeasure of chronic artificial gravity which eliminates the occupational hazards of microgravity. A European Commission directive on this subject states: “*Within the context of his responsibilities, the employer shall take the measures necessary for the safety and health protection of workers, including prevention of occupational risks and provision of information and training, as well as provision of the necessary organization and means*”(EEC_Council, 1989). Similar wording to guide national policies is used in Convention 155 of the International Labor Organization (ILO) (International Labor Organization, 1983).

There is also the other occupational hazard especially prominent when going outside the protective Earth magnetic field of the van Allen belt, i.e., solar flares and high charge and the very energetic particles from galactic cosmic rays. For the latter there are strong indications that such radiation is prone to induce e.g. malignancies or retard brain functions (Delcourt et al., 2018; Raber et al., 2019). In contrast to providing gravity to astronauts in case of the micro-gravity hazard, it is much more difficult to mitigate the impact of especially the galactic radiation. Similarly, attention to a suitable station architecture, procedures and nutritional provisions are needed as well (Bergouignan et al., 2016).

Very few papers address the ethics with respect to spaceflight. The very high-tech and heroic nature of human spaceflight would appear to be exempt from addressing labor-related, safe and healthy working environments. The one example we could find where spaceflight is related to ethical issues was actually more linked to future commercial spaceflight and possible legal issues for tourist customers (Marsh, 2006). However, the issue of ethical conduct in the working environment is very current and actually started already at the time we became aware of the deleterious effects of long duration microgravity, quite some decades ago.

Is it ethical to withhold gravity? No it is not! Career space workers as well as future space tourists should be provided with adequate levels of gravity in order to mitigate or completely abolish the microgravity-related pathologies we currently see. It is technologically feasible and financially achievable but most of all unethical not to do so.

## Author Contributions

All authors listed have made a substantial, direct and intellectual contribution to the work, and approved it for publication.

## Conflict of Interest

The authors declare that the research was conducted in the absence of any commercial or financial relationships that could be construed as a potential conflict of interest.

## References

[B1] Barger L. K. Flynn-Evans E. E. Kubey A. Walsh L. Ronda J. M. Wang W.. (2014). Prevalence of sleep deficiency and use of hypnotic drugs in astronauts before, during, and after spaceflight: an observational study. Lancet Neurol. 13, 904–912. 10.1016/S1474-4422(14)70122-X 25127232 PMC4188436

[B2] Bergouignan A. Stein T. P. Habold C. Coxam V. D. O. G. Blanc S. (2016). Towards human exploration of space: the THESEUS review series on nutrition and metabolism research priorities. NPJ Microgravity 2:16029. 10.1038/npjmgrav.2016.29 28725737 PMC5515527

[B3] Bikle D. D. Halloran B. P. (1999). The response of bone to unloading. J. Bone Miner. Metab. 17, 233–244. 10.1007/s007740050090 10575587

[B4] Delcourt C. Le Goff M. Von Hanno T. Mirshahi A. Khawaja A. P. Verhoeven V. J. M.. (2018). The decreasing prevalence of nonrefractive visual impairment in older europeans: a meta-analysis of published and unpublished data. Ophthalmology 125, 1149–1159. 10.1016/j.ophtha.2018.02.005 29548645

[B5] Dickerman R. D. Mcconathy W. J. Smith G. H. East J. W. Rudder L. (2000). Middle cerebral artery blood flow velocity in elite power athletes during maximal weight-lifting. Neurol. Res. 22, 337–340. 10.1080/01616412.2000.11740679 10874679

[B6] EEC_Council (1989). Directive 89/391/EEC of 12 June 1989 on the Introduction of Measures to Encourage Improvements in the Safety and Health of Workers at Work. Official Journal L. ECC (Brussels: EEC).

[B7] Fuller P. M. Jones T. A. Jones S. M. Fuller C. A. (2002). Neurovestibular modulation of circadian and homeostatic regulation: vestibulohypothalamic connection? Proc. Natl. Acad. Sci. U.S.A. 99, 15723–15728. 10.1073/pnas.242251499 12434016 PMC137783

[B8] Globus A. Hall T. (2017). Space settlement population rotation tolerance. NSS Space Settle. J.

[B9] Goswami N. van Loon J. Roessler A. Blaber A. P. White O. (2019). Editorial: gravitational physiology, aging and medicine. Front. Physiol. 10:1338. 10.3389/fphys.2019.01338 31708798 PMC6823592

[B10] Hall T. W. (2016). “Artificial gravity in theory and practice,” in Paper presented at the 46th International Conference on Environmental Systems (Vienna).

[B11] International Labor Organization I. (1983). “Occupational safety and health and the working environment,” in Convension # 155 ed Ilo (Geneva: International Labor Organization).

[B12] Joosten B. K. (2007). Preliminary Assessment of Artificial Gravity Impacts to Deep-Space Vehicle Design (JSC-63743). Houston, TX.

[B13] Lang T. Van Loon J. Bloomfield S. Vico L. Chopard A. Rittweger J.. (2017). Towards human exploration of space: the THESEUS review series on muscle and bone research priorities. NPJ Microgravity 3:8. 10.1038/s41526-017-0013-0 28649630 PMC5445590

[B14] Law J. Van Baalen M. Foy M. Mason S. S. Mendez C. Wear M. L.. (2014). Relationship between carbon dioxide levels and reported headaches on the international space station. J. Occup. Environ. Med. 56, 477–483. 10.1097/JOM.0000000000000158 24806559

[B15] Levasseur R. Sabatier J. P. Etard O. Denise P. Reber A. (2004). Labyrinthectomy decreases bone mineral density in the femoral metaphysis in rats. J. Vestib. Res. 14, 361–365. 15598990

[B16] Mader T. H. Gibson C. R. Pass A. F. Kramer L. A. Lee A. G. Fogarty J.. (2011). Optic disc edema, globe flattening, choroidal folds, and hyperopic shifts observed in astronauts after long-duration space flight. Ophthalmology 118, 2058–2069. 10.1016/j.ophtha.2011.06.021 21849212

[B17] Marsh M. (2006). Ethical and medical dilemmas of space tourism. Adv. Space Res. 37, 1823–1827. 10.1016/j.asr.2006.03.001

[B18] Marshall-Goebel K. Laurie S. S. Alferova I. V. Arbeille P. Aunon-Chancellor S. M. Ebert D. J.. (2019). Assessment of jugular venous blood flow stasis and thrombosis during spaceflight. JAMA Netw. Open 2:e1915011. 10.1001/jamanetworkopen.2019.15011 31722025 PMC6902784

[B19] Martin K. M. Landau D. F. Longuski J. M. (2016). Method to maintain artificial gravity during transfer maneuvers for tethered spacecraft. Acta Astronaut. 120, 138–153. 10.1016/j.actaastro.2015.11.030

[B20] National Research Council S. S. B. (2018). A Midterm Assessment of Implementation of the Decadal Survey on Life and Physical Sciences Research at NASA NAS May 2018. NAS. 29924532

[B21] Ogoh S. Marais M. Lericollais R. Denise P. Raven P. B. Normand H. (2018). Interaction between graviception and carotid baroreflex function in humans during parabolic flight-induced microgravity. J. Appl. Physiol. (1985) 125, 634–641. 10.1152/japplphysiol.00198.2018 29745800

[B22] Paloski W. H. Charles J. B. (2014). 2014 International Workshop on Research and Operational Considerations for Artificial Gravity Countermeasures, eds P. Norsk, L. Smith, R. Cromwell, J. Kugler, C. Gilbert, and D. Baumann (Hanover, MD: Editorial Board).

[B23] Raber J. Yamazaki J. Torres E. R. S. Kirchoff N. Stagaman K. Sharpton T.. (2019). Combined effects of three high-energy charged particle beams important for space flight on brain, behavioral and cognitive endpoints in B6D2F1 female and male mice. Front. Physiol. 10:179. 10.3389/fphys.2019.00179 30914962 PMC6422905

[B24] Roberts D. R. Albrecht M. H. Collins H. R. Asemani D. Chatterjee A. R. Spampinato M. V.. (2017). Effects of spaceflight on astronaut brain structure as indicated on MRI. N. Engl. J. Med. 377, 1746–1753. 10.1056/NEJMoa1705129 29091569

[B25] Stahn A. C. Werner A. Opatz O. Maggioni M. A. Steinach M. Von Ahlefeld V. W.. (2017). Increased core body temperature in astronauts during long-duration space missions. Sci. Rep. 7:16180. 10.1038/s41598-017-15560-w 29170507 PMC5701078

[B26] Stenger M. B. Tarver W. J. Brunstetter T. Gibson C. R. Laurie S. S. Lee S. (2017). Evidence Report: Risk of Spaceflight Associated Neuro-ocular Syndrome (SANS). NASA.

[B27] Stepanek J. Blue R. S. Parazynski S. (2019). Space medicine in the Era of Civilian spaceflight. N. Engl. J. Med. 380, 1053–1060. 10.1056/NEJMra1609012 30865799

[B28] Trudel G. Shafer J. Laneuville O. Ramsay T. (2019). Characterizing the effect of exposure to microgravity on anemia, more space is worse. Am. J. Hematol. 38, 293–303. 10.1002/ajh.25699 31816115

[B29] van Loon J. J. W. A. Baeyens J. P. Berte J. Blanc S. Braak L. Bok K. (2012). A large human centrifuge for exploration and exploitation research. Ann. Kinesiol. 3, 107–121.

[B30] Van Ombergen A. Jillings S. Jeurissen B. Tomilovskaya E. Rühl R. M. Rumshiskaya A.. (2018). Brain tissue-volume changes in cosmonauts. N. Engl. J. Med. 379, 1678–1680. 10.1056/NEJMc1809011 30354959

[B31] Vico L. Van Rietbergen B. Vilayphiou N. Linossier M. T. Locrelle H. Normand M. (2017). Cortical and trabecular bone microstructure did not recover at weight-bearing skeletal sites and progressively deteriorated at non-weight-bearing sites during the year following International Space Station missions. J. Bone Miner. Res. 32, 2010–2021. 10.1002/jbmr.3188 28574653

[B32] Waldrop M. M. (1982). Astronauts can't stomach zero gravity. Science 218, 1106. 10.1126/science.218.4577.1106 17752859

[B33] Yaqub F. (2015). Space travel: medicine in extremes. Lancet Respir. Med. 3, 20–21. 10.1016/S2213-2600(14)70192-4 25466547

[B34] Zhang L.-F. Hargens A. R. (2017). Spaceflight-induced intracranial hypertension and visual impairment: pathophysiology and countermeasures. Physiol. Rev. 98, 59–87. 10.1152/physrev.00017.2016 29167331

